# Development of a fast and economical genotyping protocol for bovine leukocyte adhesion deficiency (BLAD) in cattle

**DOI:** 10.1186/s40064-016-3148-7

**Published:** 2016-08-30

**Authors:** Rafeeque R. Alyethodi, Umesh Singh, Sushil Kumar, Rajib Deb, Rani Alex, Sheetal Sharma, Gyanendra S. Sengar, B. Prakash

**Affiliations:** ICAR-Central Institute for Research on Cattle, Grass Farm Road, Meerut Cantt, Meerut, UP 250001 India

**Keywords:** BLAD, T-ARMS PCR, SNP genotyping, PCR–RFLP, Frieswal

## Abstract

Fast and economical means of assaying SNP’s are important in diagnostic assays, especially when a large number of animals have to be screened for a genetic disease. This study was aimed at the development of a fast and economical screening assay for bovine leukocyte adhesion deficiency (BLAD) which is an important genetic disease of cattle industry. Four primers were designed where the outer primers amplify a 354 bp amplicon of CD18 gene carrying the polymorphism responsible for BLAD. The specifically designed inner primers in conjunction with the modified reaction mixture and cyclic conditions ensured amplification of either of wild or mutated alleles. Together with outer primers, the inner primers generated typical banding pattern in agarose gel which discriminated the normal animal against the carrier. We successfully used this protocol in 200 bulls for genotyping the BLAD allele which confirmed by sequencing, showing a cent percentage concordance. With the developed assay the need for restriction digestion or use of costly equipment viz. real time PCR was eliminated. This genotyping assay ensured fast and economical genotyping and could be adopted in every laboratory with a minimum equipment requirement of thermocycler and gel documentation system.

Holstein–Friesian (HF) semen are used extensively worldwide through Artificial Insemination (AI) during the last 4–5 decades. As a result, the HF-specific genetic diseases became diseases of economic importance in the dairy industry. In autosomal recessive disorders of cattle, carriers are more responsible for transmitting the mutant allele to the next generation as they don’t show clinical symptoms of the diseases. The situation gets dreadful if the carriers are bulls intended to use extensively in artificial breeding programs. The clinical outcomes such as recurrent bacterial infections, oral ulceration, pyorrhea, chronic pneumonia, chronic diarrhea and death in the calf hood stage are due to the inability of the leukocytes to perform cell surface adherence functions. Normally, leukocytes express β2 integrins (CD11a, b, c/CD18) glycoproteins which are mediators of the cell to cell and cell to extracellular matrix interactions (Nagahata 2004). A point mutation (adenine to guanine) at the 383rd position of the CD18 gene (Kehrli et al. 1990) causes aspartic acid to glycine substitution at 128th amino acid position (D128G; Nagahata 2004). This substitution impairs the glycoprotein leading to dysfunction of the adherence dependent functions of leukocytes. Cattle with BLAD dies before 1 year of age while the survived cows show low production and reproduction performances (Meydan et al. 2010). Advances in molecular biotechnology enable fast and reliable methods for accurate diagnosis of mutations responsible for different genetic defects. These assist breeders to identify carriers at an early stage. Different techniques viz. allele-specific PCR (CN101899511 B, patent 2010), PCR–RFLP (Natonek 2000) and real time PCR (Zhang et al. 2012) are used to identify BLAD carrier animals. But these assays are either time consuming and expensive. Therefore, a rapid and economical test for routine screening of animals for BLAD are always in demand.

The Amplification Refractory Mutation System (ARMS)-PCR (Newton et al. 1989) and tetra-primer PCR (Ye et al. 1992) detects known sequence polymorphisms. The combination of aforesaid two technique generated tetra-primer ARMS-PCR or T-ARMS technique (Ye et al. 2001). Allele-specific amplification was achieved using two outer primers and two allele-specific inner primers in a single PCR reaction mix. The deliberate mismatch introduced at position −2 from the 3′ end of the inner primers improve allele specificity. In short, in a single tube reaction, the outer forward (OF) and outer reverse (OR) primers amplify a specific amplicon of the target gene, irrespective of the allele at SNP position. The inner forward (IF) and inner reverse (IR) primers with OR and OF primers respectively generate allele-specific amplicons. These amplicons will be of different sizes, hence easily discriminated on an agarose gel as either homozygous or heterozygous. While the two outer primers (OF, OR) ensure the gene specificity and PCR efficiency, the inner outer combination (OF/IR, IF/OR) ensures the allele specificity. In the current study, we developed a tetra-primer single tube PCR-based assay for detection of BLAD carriers in the cattle. It is a fast and economical assay for the screening of BLAD carriers among cattle.

## Methods

### Sample collection

Frieswal (HF × Sahiwal cross) bull calves and young bulls reared at the bull rearing unit of ICAR-Central Institute for Research on Cattle were used for the study. Genomic DNA was isolated from whole blood by conventional phenol–chloroform method (Sambrook et al. 1989) with minor modifications. DNA was isolated from semen samples using the Guanidium thiocyanate method (Hossain et al. 1997) with modifications (unpublished data). The isolated DNA were run on an agarose gel electrophoresis (0.7 %) for quality assessment. The quantity and purity were measured by NanoDrop spectrophotometer (Thermo scientific). The DNA were kept dissolved in TE buffer (pH 8.0) at −20 °C until use.

### Tetra ARMS primer designs

Primers were designed by the original software on the website: http://cedar.genetics.soton.ac.uk. Four primers were designed viz. OF (5′-GAATAGGCGTCCTGCATCCTATCCACCA) and OR primers (5′-CTTGGGGTTTCAGGGGAAGATGGAGTAG) were used to amplify the CD18 gene, while the specially designed IF (5′-GGCCAAGGGCTACCCCATAGA) for An allele and IR primer (5′-GTAGGAGAGGTCCATCAGGTAGTACATGC) for the G allele enabled specific amplification of normal and mutant alleles. The mutation points were positioned asymmetrically with respect to the common (outer) primers so that allele-specific amplicons with different product lengths could be easily separated by standard agarose gel electrophoresis. The primer specificity is tested using the BLAST program of NCBI.

### In vitro amplification, visualization and analysis

The composition of each T-ARMS PCR reaction mix consisted of 80–100 ng of good quality genomic DNA, 0.1 μM of each primer i.e. OF (5′-GAATAGGCGTCCTGCATCCT-ATCCACCA), OR (5′-CTTGGGGTTTCAGGGGAAGATGGAGTAG), IF (5′-GGCCAAGG-GCTACCCCATAGA) and IR (5′-GTAGGAGAGGTCCATCAGGTAGTAC-ATGC), 200 μM of each dNTP and 1 U Taq DNA polymerase with buffer containing 1.5 mM MgCl_2_. An additional 0.25 μl of 50 mM MgCl_2_ was added to the reaction mix to make the final concentration to 2 mM. The mix is further supplemented with 1.25 μl DMSO (5 %) and the final reaction volume of 25 μl was made up with nuclease-free water (Ambion). After an initial denaturation at 94 °C for 5 min, the PCR were set for 35 cycles consisting of a denaturation step at 94 °C for 30 s, an annealing step at 55 °C for 45 s, and an extension step at 72 °C for 35 s. Lastly, a final extension 72 °C for 10 min was provided. The generated amplicons were separated by 1.5 % agarose gel and visualized under UV light by AlphaImager gel documentation system. The carrier and normal allele are differentiated by checking the amplicon sizes in reference with size markers.

### Validation of the assay

Simple PCR were performed in a final reaction volume of 10 μl. Each PCR mix consisted of 50–100 ng of good quality genomic DNA, 0.1 μM of each primer i.e. Forward primer 5′-GAATAGGCGTCCTGCATCCTATCCACCA and Reverse primer 5′-CTTGGGGTTTCAG-GGGAAGATGGAGTAG, 200 μM of each dNTP and 1 U Taq DNA polymerase. After an initial denaturation at 94 °C for 5 min, the PCR set for 35 cycles consisting of a denaturation step at 94 °C for 30 s, an annealing step at 60 °C for 45 s, an extension step at 72 °C for 35 s. Lastly, a final extension 72 °C for 10 min was provided. 2 μl of amplified product were run on 2 % agarose gel electrophoresis and amplification was assessed. A volume of 8 μl of PCR products was digested with Taq1 in a final volume of 15 µl. The digestion cocktail contained 5–10 Units of enzyme. The digested products were separated on 3 % agarose gel and analyzed by AlphaImager EP gel doc system. For sequencing of a mutant allele of BLAD, 354 bp band obtained after gel separation of digested product were eluted and purified by gel extraction kit (Sigma, Aldrich) from the carrier animals. For sequencing of the wild type allele of BLAD, PCR products from the normal animals were utilized. Both were sequenced using ABI 3100 (Applied Biosystems, USA) Automated DNA Sequencer. Samples were sequenced with forward as well as the reverse primer to confirm the findings. The raw sequence data were edited using Chromas (Ver. 1.45, http://www.technelysium.com) and the variation were confirmed by manual inspection of chromatograms. The BLAST algorithm was used to analyze the mutation in the generated sequence against the sequences in the NCBI Gen bank databases.

### Pedigree analysis

To trace the inheritance, the bull pedigree for the carrier bull calves were analyzed using PROGENY9 (V. 1.32).

## Results and discussion

**Fig. 1:**
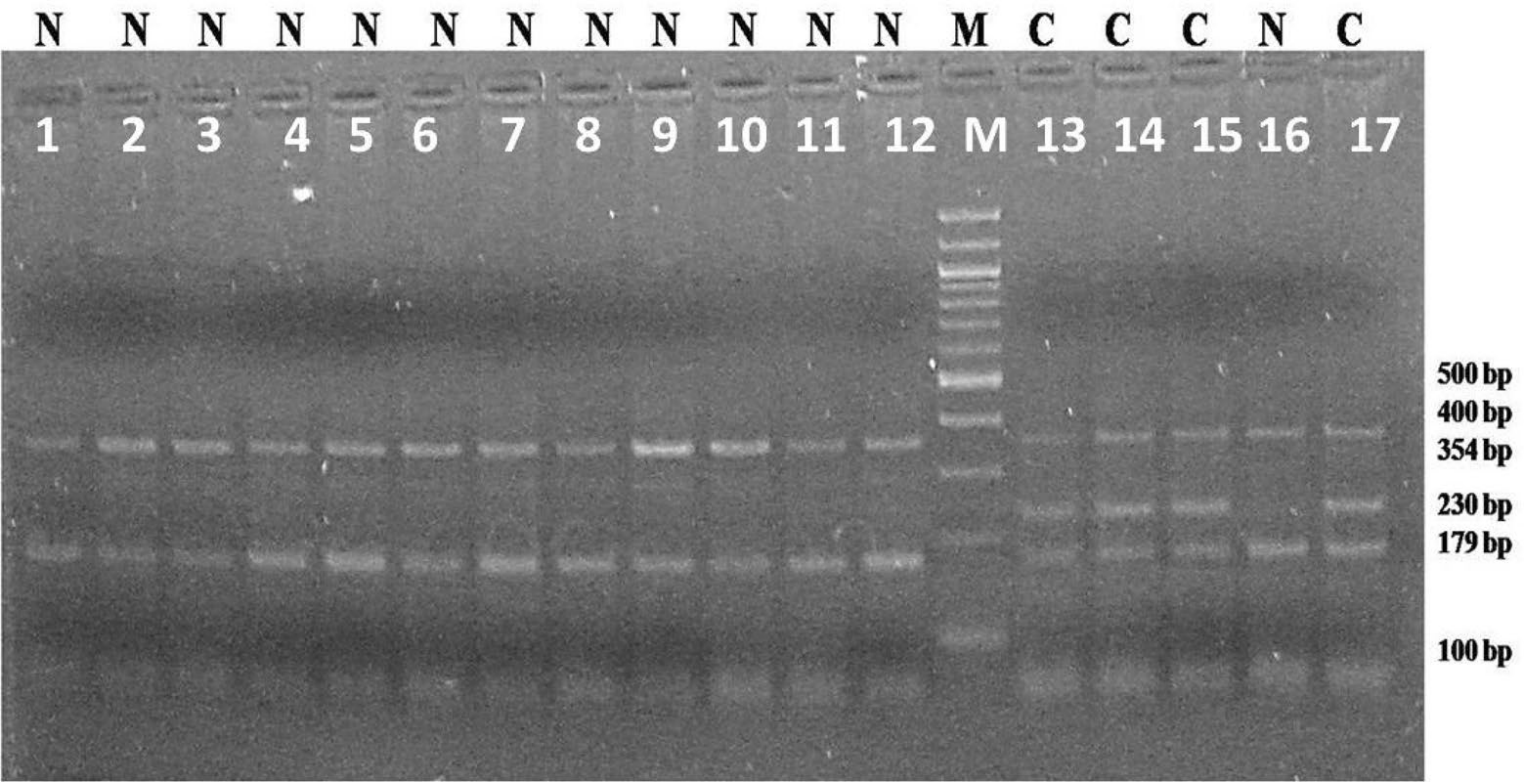
T-ARMS-PCR based genotyping of BLAD. The outer primers (OF and OR) amplified a 354 bp product. The inner forward primer can detect normal allele (adenine) at position 383rd of CD18 gene with an amplicon size of 179 bp while inner reverse primer can detect mutated allele (guanine) at the same position with an amplicon size of 230 bp (*lanes 13, 14, 15* and *17*)

Using the developed Tetra-ARMS-PCR assay, we genotyped SNP at position 383 of the CD18 gene, responsible for the BLAD. The outer primers (OF and OR) amplified a 354 bp product. The inner forward primer detected normal allele (Adenine) at position 383 of CD18 gene with an amplicon size of 179 bp while inner reverse primer detected mutated allele (Guanine) at the same position with an amplicon size of 230 bp (Fig. 1). In agarose gel, normal animals showed a 354 and 179 bp amplicons while the carriers showed an additional band of 230 bp.

T-ARMS PCR needs extensive optimization compared to simple PCR. Various parameters such as annealing temperature, primer concentration, Inner to outer primer ratio, MgCl_2_ and dNTPs concentration, Taq polymerase concentration etc., needs to be optimized. The DNA extraction method affects the outcome of T-ARMS PCR (Medrano and de Oliveira 2014). Various authors have undertaken different approaches to overcome these difficulties and successfully generated T-ARMS genotyping (Medrano and de Oliveira 2014; Fonseca et al. 2013; Ahlawat et al. 2014; Singh et al. 2014).

Allele-specific amplification in T-ARMS PCR is greatly influenced by the melting temperature (Chiapparino et al. 2004). In this study, the annealing temperature of 60 °C found better except for the lower amplicon of 179 bp which showed a low-intensity band in agarose gel electrophoresis giving unbalanced amplification among different bands viz. 354, 230, and 179 bp. The used primers showed varying GC content ranging from 51.7 (Inner Reverse) to 61.9 % (Inner Forward). It causes unbalanced amplification in complex PCR assays. It is because GC-rich sequences in conjunction with high Tm leads to a formation of stable secondary structures in the primers, which reduces the PCR efficiency by serving as termination or stop sites (McDowell et al. 1998). Moreover, these stop sites causes an erroneous incorporation of an extra base which is highly resistant to elongation in T-ARMS assays (Medrano and de Oliveira 2014). We used DMSO and Betaine as PCR enhancers. DMSO bind and disrupt cytosine base pairing with guanine which brings downs the melting temperature of the primer. In other hand betaine, an isostabilizing agent, equalizes the contribution of GC- and AT-base pairing and improve the stability of the DNA duplex (Frackman et al. 1998; Varadaraj and Skinner 1994). Use of betaine at a different concentration ranging from 0.1 to 1.0 M didn’t yield any significant amplification (data not showed). Similar to our observation, betaine showed to be inefficient for enhancing the amplification of GC-rich regions (Medrano and de Oliveira 2014). We tested DMSO at 5, 8 and 10 % level. A 5 % DMSO level in the PCR mix enhanced PCR amplification at a lower annealing temperature of 55 °C. DMSO in conjunction with MgCl_2_ (2 mM) generated balanced amplification of all amplicons and eliminated the nonspecific bands.

The quality of DNA varies with the method of extraction. An OD 260/280 ratio of 1.8–2 and OD 260/230 of 2 expected for good quality extracted DNA. Extraction of blood gDNA using phenol–chloroform method yielded DNA from all the samples within expected ratio. The sperm DNA extracted by lysis using guanidium thiocyanate showed similar OD 260/280 ratios but lower 260/230 ratios. The developed T-ARMS PCR assay found unaffected by the both extraction protocols. We tested primer concentrations from 0.1 to 0.5 μM and an inner to the outer primer ratio of 1:1 to 10:1. A primer concentration of 0.1 μM at 1:1 ratio give sufficiently good amplification compared to other ratios and concentrations. The final MgCl_2_ concentration tried ranged 1.5–4 mM of which 2 mM with DMSO at 5 % level gave balanced amplification of all bands.

**Fig. 2:**
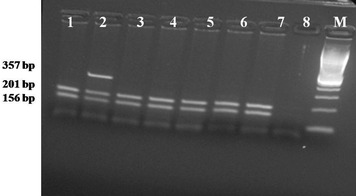
Illustration of BLAD genotypes pattern after PCR–RFLP on agarose gels. *Lanes* are numbered from *left* to *right*. The marker used is 100 bp. *Lane 2* show digestion pattern for heterozygous animals with 3 bands of 357, 201 and 156 bp while other lanes (*lanes 1, 3, 4, 5, 6* and *7*) show normal homozygote pattern with 201 and 156 bp bands

**Fig. 3:**
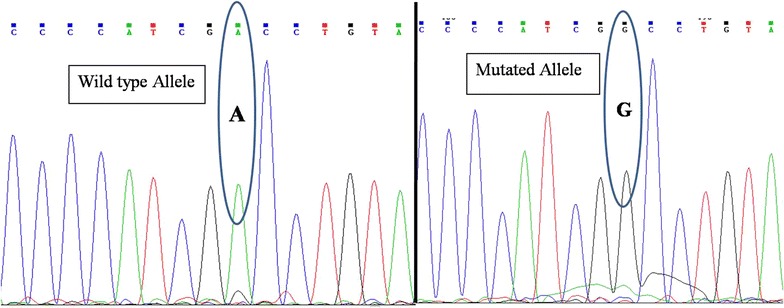
Chromatograph analysis showed the presence guanine mutated allele instead of adenine in the wild type allele

We compared the genotyping results of developed tetra-primer ARMS-PCR with the existing PCR–RFLP genotyping protocol and further by sequencing. A 100 % concordance between the results of the two methods indicate both high sensitivity and specificity of the T-ARMS PCR assay. In PCR–RFLP assay, normal BLAD allele produced two fragments of 201 and 156 bp while BLAD carriers produced three fragments of 354, 201 and 153 bp (Fig. 2). In the current study, seven bulls were heterozygous for BLAD allele showing a carrier prevalence of 4.6 %. The chromatograph analysis of PCR products sequencing confirmed the presence of a mutated allele (Fig. 3). We also carried the pedigree analysis of all the carrier animals which traced back to the imported semen of exotic HF. The incidence of BLAD was very high (23 %) in earlier studies (Shuster et al. 1992). Due to regular screening, the incidences are reducing the world over (Meydan et al. 2010; Li et al. 2011; Rajesh et al. 2007; Arpita et al. 2012; Patel et al. 2011). Similar to world scenario, the prevalence of carrier and affected animals in Karan Fries strains of India (Holstein and Tharparkar cross) are 3.64 and 1.84 %, respectively (Yathish et al. 2010).

## Conclusions

For the first time, tetra-primer ARMS-PCR were used for the genotyping for bovine leukocyte adhesion deficiency (BLAD) in cattle. The developed assay matched cent percent with the standard PCR–RFLP results. PCR–RFLP needs a length digestion step before genotyping while real time PCR-based genotyping require costly equipment and reagents. The developed protocol is a faster and cheaper alternative assay for PCR–RFLP and real time-based assays. Development of fast assays for genotyping of other genetic diseases of cattle can fasten the screening process and avoid the cost of rearing diseased animals in their population.
